# Mapping defect distribution in transparent single-walled carbon nanotube film with electrical resistance tomography

**DOI:** 10.1038/s41598-025-29839-w

**Published:** 2025-12-18

**Authors:** Keiya Minakawa, Taiki Nakada, Reiji Kaneko, Takashi Ikuno

**Affiliations:** Department of Applied Electronics, Graduate School of Advanced Engineering, Tokyo University of Science, Katsushika, Tokyo, 125-8585 Japan

**Keywords:** Materials science, Nanoscience and technology, Optics and photonics, Physics

## Abstract

We extend the application of electrical resistance tomography (ERT) to visualize *I*_G_/*I*_D_ distribution in transparent single-walled carbon nanotube (SWCNT) thin films. By establishing the correlation between disorder and resistance through experimental measurements, we successfully converted ERT-reconstructed conductivity distributions into *I*_G_/*I*_D_ maps. This approach enabled the detection of a defect region with an *I*_G_/*I*_D_ ratio of approximately 15 embedded in a pristine SWCNT film (*I*_G_/*I*_D_ = 18.1), corresponding to a minimum detectable resistance ratio (*R*/*R*_0_) of 1.59. Moreover, in a sample containing two defect regions introduced by plasma irradiation at 20 and 40 W, ERT successfully distinguished them despite the small difference in *I*_G_/*I*_D_ ratio (1.1). Simulation studies revealed that positional deviations in reconstructed images can be attributed to electrode misalignment, contact resistance variation, and plasma-induced defect expansion. These results demonstrate the feasibility of ERT as a non-destructive, high-resolution technique for evaluating disorder variations in conductive thin films. Furthermore, due to its conductivity-based imaging principle, the method is applicable to other materials such as graphene, indium tin oxide (ITO), and metal nanowire networks, offering promise for real-time monitoring and quality assurance in large-area optoelectronic device manufacturing.

**Supplementary Information:**

The online version contains supplementary material available at 10.1038/s41598-025-29839-w.

## Introduction

Single-walled carbon nanotube (SWCNT) thin films have attracted attention as one of the key materials for various optoelectronic devices such as solar cells, touch panels, and organic light-emitting diode (OLED) display because of their high transparency, excellent electrical conductivity, and mechanical strength^1–5^. In these devices, microscopic defects including atomic voids, *sp*^2^-to-*sp*^3^ conversion, contamination, and surface functionalization of oxygen-containing groups can adversely affect performance. Therefore, real-time monitoring of the disorder and defect distribution across the film surface is essential for quality control^3^.

Traditionally, Raman mapping has been employed to detect microscopic defects in SWCNT thin films^1,6^. This technique involves scanning a focused laser across the sample surface to generate two-dimensional disorder maps, offering micrometer-scale spatial resolution^7^. While Raman mapping provides highly localized and precise structural information, its point-by-point nature limits its throughput for large-area evaluation, particularly in manufacturing contexts.

To complement this technique, we focus on electrical resistance tomography (ERT), a non-destructive imaging technique that reconstructs internal conductivity distributions across a wide area^8–10^. In this experiment, a constant current was injected between a pair of adjacent electrodes, while the potential differences between all other adjacent electrode pairs were measured. ERT can be applied to the non-destructive testing of large-scale samples such as the human body and buildings^8,11–13^. Inspired by these reports, we explored the application of ERT to large-area transparent SWCNT thin films. In our ERT configuration, one scan (40 voltage measurements) takes approximately 1–2 s. In contrast, to obtain a Raman map of comparable spatial coverage and voxel size (≈ 1 mm resolution across a 14 mm-diameter film), about 2000 spectra must be collected, requiring roughly 15–100 min depending on the exposure time per point (0.5–3 s). Although the total measurement times can be comparable when including electrode fabrication (~ 13 min per sample), ERT provides direct visualization of the conductivity distribution, enabling detection of functional defects such as disrupted current paths that cannot be captured by Raman spectroscopy. Therefore, Raman and ERT serve as complementary, rather than competing, characterization techniques.

While previous studies^14–17^ have demonstrated the use of ERT to visualize electrical conductivity distributions and detect defects in various materials, the correlation between the conductivity distributions visualized by ERT and the microstructural properties such as local disorder has not yet been clarified. In addition, since multiple electrodes are placed along the edge of the sample for ERT measurements, the measured potential data contain errors due to variations in contact resistance at each electrode^18–23^.

In this study, we extend the application of ERT to visualize defects caused by increased disorder in transparent SWCNT thin films. In addition, the effect of the interfaces between multiple electrodes and the SWCNT thin film on the position of defects was investigated through simulations. A transparent SWCNT thin film with an *I*_G_/*I*_D_ ratio of 18.1 and an optical transmittance of 84.9% at 550 nm was fabricated. Using ERT, high disorder regions were distinguished and visualized, specifically the region with an *I*_G_/*I*_D_ ratio of 11.6 after plasma irradiation at 20 W and the region with an *I*_G_/*I*_D_ ratio of 7.3 after plasma irradiation at 40 W. Furthermore, using simulations, we investigated three factors that affect the position of defects in the reconstructed images: the misalignment between the center of the circular SWCNT film and the center of the eight electrodes, variations in the contact resistances of the eight electrodes, and the enlargement of defects caused by plasma penetration between the metal mask and the SWCNT thin film during plasma irradiation.

## Method

### Film fabrication and defect introduction

**Fig. 1:**
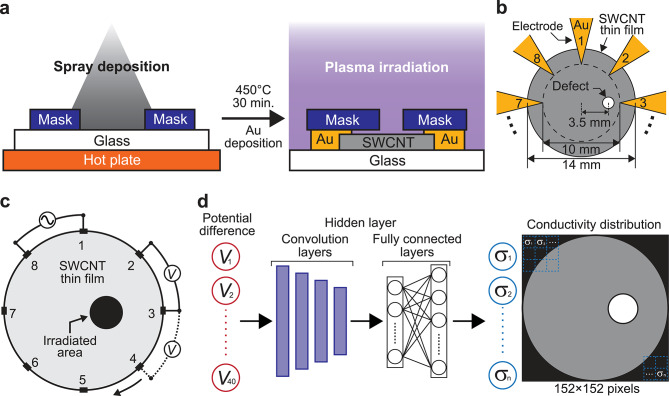
(**a**) Schematic illustration of the fabrication process of SWCNT thin films and the defect formation induced by plasma irradiation. (**b**) SWCNT thin film with Au-deposited electrodes designed for ERT measurements. (**c**) Typical setup for ERT measurements. (**d**) Neural network structure used for ERT image reconstruction.

Figure 1a shows a schematic illustration of the SWCNT film fabrication process. A soda-lime glass substrate (thickness: 1 mm; size: 2 × 2 cm; Tokyo Glass Co., Ltd., Japan) covered by a metal mask with a 14 mm diameter hole was placed on a hot plate (HP-1SA, AS ONE Corporation, Osaka, Japan) at 110 °C. Then, an SWCNT thin film was deposited on the substrate using a custom-made spraying machine^24^. The SWCNT thin film was circular with a diameter of 14 mm. For the spraying deposition, as the ink, mixture of 0.1 mL of aqueous solution (0.2 wt%, EC-DH, Meiji Nano Carbon Co., Ltd., Japan) diluted by 5 mL of deionized water, and 25 µL of a 16 wt% surfactant solution composed of sodium alkyl ether sulfate and fatty acid alkanolamide (031 − 15, Saraya Co., Ltd., Japan)^25^ was used. Before spraying, the ink was homogenized using an ultrasonic homogenizer (FS-300 N, Shenzhen XinzhiBang Inst & Eqpt. Co., Ltd., China) at 150 W power for 90 s. The fabricated SWCNT films were annealed at 450 °C for 30 min. using an infrared radiation furnace (MILA-3000, ULVAC, Inc., Japan), followed by deposition of patterned gold (Au) thin film as for the electrode of ERT measurement using a vacuum evaporator (VPC410, ULVAC, Japan). Eight triangular electrodes with their vertices pointing toward the center were fabricated. The center of the electrodes was aligned with the center of the circular SWCNT thin film as shown in Fig. 1b. The diameter of the circle touching the vertices was 10 mm.

The right side of Fig. 1a shows a fabrication method of defects in SWCNT thin films. The defect was fabricated by capacitively coupled plasma using a gas plasma reactor (PR200, Yamato Scientific Co., Ltd., Japan) with air as plasma source. A metal mask with circular the opening area the diameter of 1.5 mm was placed such that the center of the circular defect region was positioned 3.5 mm away from the center of the SWCNT thin film, along the line positioning toward the vertex of electrode 3. Then plasma irradiation was performed. The plasma irradiation power was varied from 10 to 40 W. The irradiation time was 10 s. In addition for another sample, plasma irradiation was applied to two different locations on the thin film surface. Detailed information about the sample will be provided later.

### Optical and electrical characterization

Optical transmittance and Raman spectra of the fabricated SWCNT thin films were measured using ultra violet-visible spectroscopy (V-770, JASCO, Tokyo, Japan) and micro-Raman spectrometer (inVia Reflex, Renishaw plc., UK) with the wavelength of 532 nm, respectively.

To experimentally determine the conductivity values corresponding to plasma-irradiated SWCNT thin films, strip-shaped samples with a thickness of 100 nm and dimensions of 3 × 10 mm were fabricated using the same method described above. Plasma irradiation was applied to 3 × 3 mm area at the center of each film at powers ranging from 5 to 40 W for 10 s. Silver (Ag) paste (DOTITE D-500, FUJIKURA KASEI Co., Ltd.) was applied to both ends of the strip-shaped film as electrodes. The electrical resistance along the longitudinal direction was measured using current-voltage (*I*-*V*) measurements with a source meter (2612 A, Keithley, OH, USA) and a probe station (MBP-55-TRI, APOLLO WAVE, Osaka, Japan). The resistance *R*_0_ of the strip-shaped film before plasma irradiation and the resistance *R* after plasma irradiation were measured. Three samples were characterized for each plasma irradiation power, and the average relative resistance change (*R*/*R*_0_) was calculated. The conductivity of each film was then estimated on the measured resistance values. The disorder of the irradiated area was measured as a function of plasma irradiation power.

### ERT measurement and data processing

Figure 1c shows a schematic illustration of a typical ERT measurement setup. We adopted an 8-electrode array symmetrically spaced around the film perimeter. Compared with a 16-electrode configuration, this choice reduces the number of voltage measurements from 208 to 40 (given by *N*(*N*–3) for *N* electrodes), enabling faster acquisition and simpler handling at the cost of some spatial resolution^26^. It has also been reported that asymmetrical electrode placement reduces reconstruction accuracy^27^. The total number of potential difference data points was 40. For the ERT measurement, we used a custom-built apparatus developed by our group^12,28^. The injected current value and frequency were 0.5 mA and 1 kHz, respectively. When a constant current was injected between one pair of adjacent electrodes, the potential differences were measured between all other adjacent electrode pairs. Since the measurements were conducted at 1 kHz, where the imaginary component of impedance in SWCNT thin films is negligible^29^, the reactance was assumed to be negligible.

### Machine learning-based image reconstruction

From the potential difference data, relative conductivity distributions were obtained using a one-dimensional convolutional neural network (1D-CNN)-based machine learning (ML) method^12,30^. Figure 1d shows the neural network architecture used for image reconstruction in ERT. The input layer consisted of 40 data points corresponding to potential differences, and the output layer consisted of 23,104 data points representing a 152 × 152 pixel conductivity distribution. The loss function, optimizer, and batch size were set to mean square error, Adam optimizer, and 32, respectively. The model was developed using the Keras library with Python 3.10.

A training dataset, consisting of pairs of potential data and corresponding conductivity distribution images, was generated by solving the forward problem using finite element method (FEM) simulations in COMSOL Multiphysics 6.2. In this study, the specimen had a known circular geometry. Therefore, the ML model was trained using datasets generated for a circular specimen geometry. In principle, the method can also be applied to specimens with non-circular geometries by generating training datasets that reflect their actual boundary shapes. As the conductivity distribution images, 76,992 synthetic samples were created by assuming a two-dimensional film with negligible thickness and a diameter of 14 mm, representing the SWCNT thin film, with a uniform conductivity of s_0_ = 3333 S/m. In each sample, a single circular defect with a conductivity s lower than s_0_, with the diameter ranging from 0.25 to 6.0 mm, was placed at a random position within the film. The training datasets were generated by varying the conductivity, size, and position of circular defects, resulting in a total of 76,992 samples. Specifically, four conductivities (1125, 490, 416, and 309 S/m), corresponding to SWCNT films subjected to plasma irradiation at 10, 20, 30, and 40 W, respectively, were used. These values were obtained from the experimental measurements described earlier, and the detailed results will be presented later. The ML model was trained using these datasets, and the number of epochs was set to 500. By increasing the number of training datasets that contain various defect sizes and shapes, a more generalized ML model can be developed, which would enable the reconstruction of multiple defects and those with different sizes. Although only circular defects were used for training in this study, the learning model can be extended to visualize non-circular defect geometries by preparing corresponding training datasets. Figure S1 shows reconstruction results for non-circular defects obtained using a learning model trained with datasets containing circular, square, and triangular defect geometries.

As an additional training dataset, 14,496 synthetic samples were also created, each containing two circular defects with conductivities of 490 and 309 S/m, corresponding to plasma irradiation powers of 20 and 40 W, respectively, within a single SWCNT thin film. The ML model was trained using these datasets, and the number of epochs was set to 100. To further explore the generalization capability of the ML-based ERT method, additional simulations were performed. The ML model created using training datasets containing one, two, and three defect regions visualized conductivity distributions that distinguished one, two, and three defect regions. However, the ML model did not visualize defect larger than the maximum size present in the training dataset. These results demonstrate that our ML-assisted ERT method can reconstruct conductivity distributions that closely correspond to the features within the training dataset.

As mentioned earlier, the 40 potential difference data points obtained from the ERT measurements were normalized by the minimum value among them. These normalized data were then input into the trained ML models to obtain the conductivity distributions. Subsequently, the relative conductivity distributions were visualized based on the conductivity distributions.

## Results and discussion

**Fig. 2:**
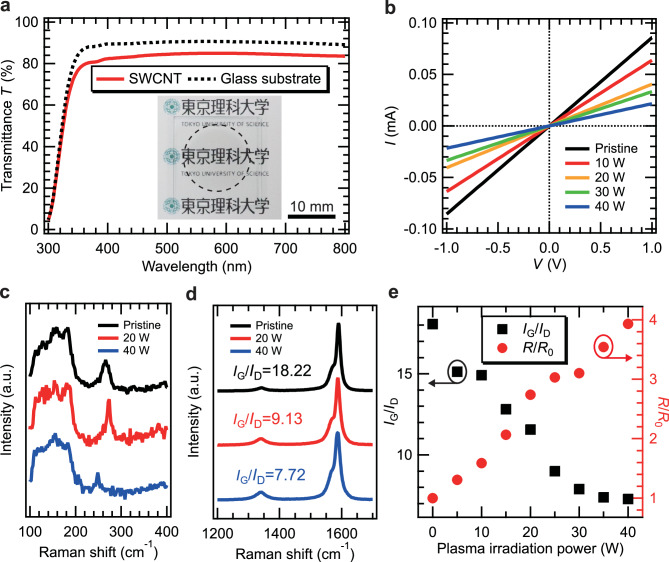
(**a**) Optical transmittance spectra of the fabricated SWCNT thin film. Inset: Photograph of the fabricated SWCNT thin film. (**b**) *I*–*V* characteristics and Raman spectra of the SWCNT thin films for (**c**) RBM region and (**d**) the range 1200 to 1700 cm^− 1^, as a function of plasma irradiation power. (**e**) Correlation between the *I*_G_/*I*_D_ ratio and *R*/*R*_0_ of the SWCNT thin film as a function of plasma irradiation power.

Figure 2a shows the optical transmittance of the samples as a function of wavelength. The inset of Fig. 2a displays a photograph of the fabricated SWCNT thin film. The SWCNT was spray-deposited within the circular dotted line. The background text beneath the transparent thin film was clearly visible to the naked eye. At 550 nm, the transmittance of the bare glass substrate and the fabricated sample were 90.6% and 84.9%, respectively. The defect distribution was difficult to visualize optically owing to an absorption of 5.8% at 550 nm. This absorbance was estimated from a transmittance of 84.9% and a reflectance of 9.3%, as shown in Fig. S2 at the same wavelength. Based on previous reports on the transmittance of SWCNT thin films as a function of thickness, the SWCNT thin film we fabricated was estimated to have a thickness of approximately 10 to 20 nm ^31^.

Figure 2b shows the *I*–*V* characteristics of fabricated SWCNT thin film sample as a function of plasma irradiation power. The *I*–*V* curves were linear, indicating ohmic contacts between the electrodes and SWCNT film. As the plasma irradiation power increased, the slope of the *I*–*V* characteristics decreased, indicating an increase in resistance. The resistance *R*_0_ of the pristine SWCNT thin film was 1.17 kΩ/cm, while the resistance *R* of the SWCNT thin film irradiated with 40 W plasma was 4.50 kΩ/cm. Therefore, the relative change in resistance *R*/*R*_0_ at a plasma power of 40 W was 3.85.

Figure 2c and d show the Raman spectra of SWCNT thin films as a function of plasma irradiation power. From the spectra, originating from radial breathing mode (RBM)^32^ shown in Fig. 2c, the diameters of SWCNTs in the film were estimated. Since broad peaks ranging from 100 to 200 cm⁻¹ and sharp peaks ranging from 240 to 260 cm⁻¹ were observed in all samples, the diameters were suggested to range from 0.9 to 2.3 nm^33^.

In Fig. 2d, two peaks around 1350 cm^− 1^ and 1580 cm^− 1^, corresponding to D-band and G-band, were observed. The intensities of D-band (*I*_D_) and G-band (*I*_G_) reflect the degree of defects in the CNTs and graphitic nature of *sp*^2^ bonds^32^, respectively. Therefore, *I*_G_/*I*_D_ ratio calculated from peak values indicates the disorder of CNTs. The *I*_G_/*I*_D_ ratios of the SWCNT thin films in the pristine, 20 W irradiated, and 40 W irradiated areas were 18.2, 9.1, and 7.7, respectively, indicating that the disorder of the SWCNT thin films increased with increasing plasma irradiation power.

Figure 2e shows the correlation between the *I*_G_/*I*_D_ ratio and *R*/*R*_0_ as a function of plasma irradiation power. As the plasma irradiation power increased, the *I*_G_/*I*_D_ ratio decreased while *R*/*R*_0_ increased. This suggests that the conductivity of the SWCNTs decreased due to increased disorder^7^. Above 30 W, the *I*_G_/*I*_D_ ratio remained constant at approximately 7, while *R*/*R*₀ continued to increase. This indicates that the disorder of the SWCNT thin film remained constant, but the decrease in conductivity may be attributed to a decrease in film thickness.

**Fig. 3:**
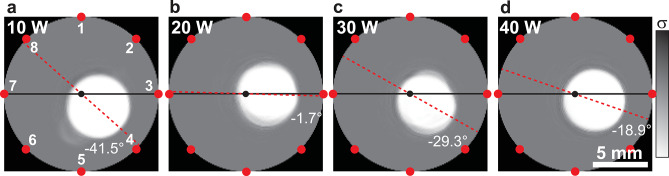
Reconstructed conductivity of samples with defects fabricated by plasma irradiation power at (**a**) 10, (**b**) 20, (**c**) 30, and (**d**) 40 W.

Next, ERT measurements were performed for the samples in which plasma irradiation at 10–40 W was applied to a circular region with a diameter of 1.5 mm, located approximately 3.5 mm to the right of the center of the SWCNT thin film. Figure 3 shows the reconstructed images of defects created by plasma irradiation. Figures 3a–d show the reconstructed images obtained using ML models trained on datasets with one circle defect. Defects were successfully visualized at all plasma irradiation powers. Even at an *R*/*R*_0_ value of 1.59, corresponding to the 10 W condition, the defect was clearly identifiable. Although the defects were all located on the right half of the circular film, the reconstructed defect regions appeared at different positions depending on the sample.

The black line connects electrodes 3 and 7 in the reconstructed image, and the red dotted line is drawn through the centroid of the white defect region and the midpoint of the black line. The rotation angle was defined as the angle between the black line and the red line, with counterclockwise rotation taken as positive. Based on this definition, the rotation angles in Figs. 3a–d were − 41.5°, − 1.7°, − 29.3°, and − 18.9°, respectively. The reconstructed defect centers in Figs. 3a–d were shifted by 1.6, 1.4, 1.8, and 1.6 mm to the left from the center of the mask opening area, and their reconstructed diameters were 5.6, 5.5, 5.4, and 5.6 mm, respectively. The comparison between the measured and simulated potential spectra is discussed in Fig. S3, confirming that the reconstructed defects were overestimated in size. In addition, plasma penetration between the mask and the SWCNT thin film may have enlarged the actual defect region beyond the 1.5 mm mask opening diameter. The slight differences in the reconstructed defect positions and orientations are mainly attributed not only to variations in contact resistance between the SWCNT film and the electrodes but also to enlargement of the defect region caused by plasma penetration beneath the mask. To verify that these deviations did not originate from the ML model itself, we tested the model using ideal potential data obtained from FEM simulations. The trained model accurately reproduced the defect size (11.4–16.1 mm for a true value of 15 mm), position (3.6 mm from the center; true = 3.5 mm), and orientation (0°), confirming that it had sufficient reconstruction accuracy. Therefore, the discrepancies observed in Fig. 3 are considered to result mainly from experimental factors, rather than from limitations of the reconstruction algorithm.

**Fig. 4:**
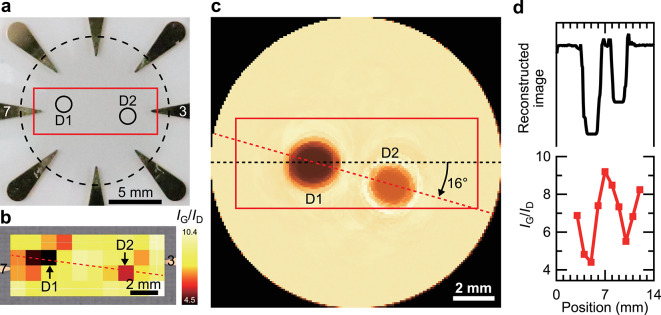
(**a**) Photographs and (**b**) Raman mapping of SWCNT thin films. (**c**) Conductivity distribution reconstructed using the ERT. (**d**) Line profiles of the conductivity distribution reconstructed by ERT and the *I*_G_/*I*_D_ ratio obtained from Raman mapping.

To extend the analysis to multiple-defect cases, we visualized two defects in an SWCNT film. Figure 4a shows photographs of samples in which the defects were created by plasma irradiation at different powers. The defects were not visually distinguishable to the naked eye. A metal mask with a 1.5 mm diameter hole was first placed 3 mm to the left of the film center, and plasma irradiation at 40 W for 10 s was applied to create the first defect (D1). A second mask was placed at the point-symmetric position, and plasma irradiation at 20 W for 10 s was applied to create the second defect (D2). The line connecting the centers of D1 and D2 was rotated by 10° relative to the line connecting the vertices of electrodes 3 and 7.

Figure 4b shows the Raman mapping of the red rectangle indicated in Fig. 4a, with a measurement resolution of 1 mm. The average *I*_G_/*I*_D_ ratios for D1 and D2 were 4.4 and 5.5, respectively, both lower than those of the surrounding regions. These results indicate that the disorder of the SWCNT thin films increased with increasing plasma power, which is consistent with the trend observed in Fig. 2e.

Figure 4c shows the reconstructed conductivity distribution obtained using ERT. The reconstruction employed the ML model described above, which was trained on a dataset comprising an SWCNT thin film and two defects (D1 and D2), with conductivities of 3333, 309, and 416 S/m, respectively. To evaluate the measurement precision, three samples were measured for each plasma irradiation power. The standard errors of the relative resistance change (*R/R*_0_) were calculated and converted into conductivity uncertainty. The relative standard errors of conductivity were 13% for 20 W and 4% for 40 W, respectively. In the reconstructed image, D1 and D2 appear as approximately circular regions. A red dotted line was drawn connecting the centroids of D1 and D2, representing the defect axis. The center of this red line was displaced by approximately 0.8 mm from the geometric center of the sample, and it was rotated by 16° relative to the line connecting electrodes 3 and 7. These displacements may be attributed to interface effects between the SWCNT film and the electrodes, or to asymmetries in the local conductivity distribution.

Figure 4d shows the line profiles of the red dotted line from the Raman mapping of the *I*_G_/*I*_D_ ratio and the conductivity distribution image reconstructed using the ERT. The Raman mapping line profiles showed that the D1 and D2 regions, with average *I*_G_/*I*_D_ ratios of 4.4 and 5.5, were visualized with relatively different disorders, while the ERT successfully distinguished these regions by their distinct conductivity values. The reason why the *I*_G_/*I*_D_ values were different from those shown in Fig. 2e might be due to that the sizes of opening areas of the metal masks during plasma irradiation were different. When the mask opening was small, energetic species generated at the edge of mask affected the entire area of SWCNT film. As a result, the disorder of the two defective areas was higher than that of the single defective area shown in Fig. 2e. In addition, another reason might be plasma penetration between the mask and film. With increasing the number of plasma irradiation process, damage might be increased if the plasma was penetrated.

From Fig. 2e, the *R*/*R*₀ values for the region irradiated at 40 W (D1) and the region irradiated at 20 W (D2) were 3.93 and 2.74, respectively, indicating that a 30% change in resistance was distinguishable and visualizable. The resistance variation might be attributed to changes in resistivity caused by increased disorder and reduced thickness of the SWCNT thin film. The ERT enabled visualization of the disorder distribution. However, the defects in the reconstructed distribution were shifted inward and rotated by 6°.

Previously, various methods have been proposed to eliminate reconstruction errors caused by contact resistance^20^, including voltage data correction for varying contact resistance^18^, detection of faulty electrodes^22^, and reduction of contact resistance between skin and electrodes by applying a contact agent to the electrode belt^21^. However, the effect of contact resistance between thin-film devices and electrode thin films has not been fully clarified.

To investigate the causes of these positional deviations, we considered three possible contributing factors: (1) misalignment between the center of the SWCNT thin film and the electrode array, (2) variations in contact resistance at the electrode–film interface, and (3) enlargement of defects due to plasma penetration beneath the metal mask. A detailed analysis of factor (1) is provided in the supplemental information. The following sections focus on simulation-based evaluations of factors (2) and (3).

In this study, the positional deviation of reconstructed defects was investigated through simulations based on three factors. One of the factors is the misalignment between the center of the circular SWCNT thin film and the center of the line connecting electrodes 3 and 7. This misalignment alters the boundary conditions of the forward problem, leading to asymmetric electric field distributions that cannot be corrected by simple translational alignment. The details are shown in the supplemental information.

**Fig. 5:**
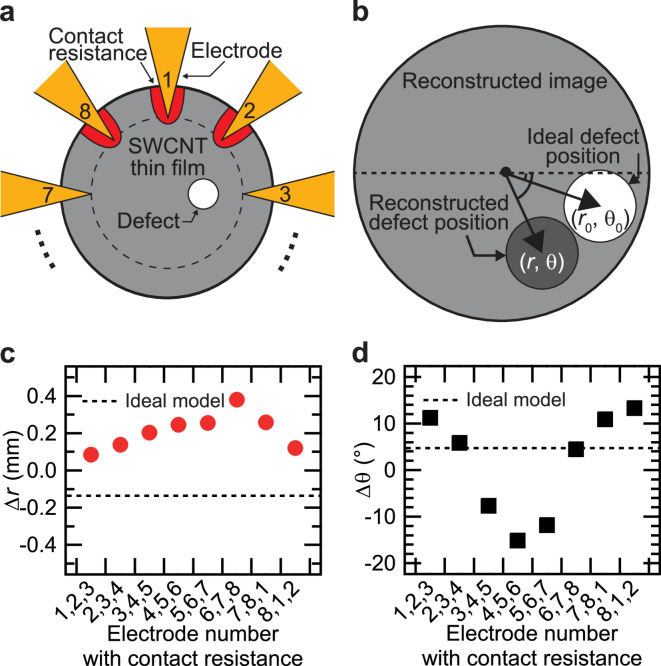
(**a**) Schematic illustration of one of the simulation model with three low conductivity regions and (**b**) the defect position estimation method. (**c**) $\:\varDelta\:r$ and (**d**) $\:\varDelta\:\theta\:$ as a function of the electrode number with contact resistance.

The second factor is variations in the contact resistances of the eight electrodes. Figure 5a shows schematic illustration of the simulation models. The red region is the low conductivity area which represents the contact resistance. The influence of contact resistance was modeled by introducing low conductivity regions near the electrodes in the simulation model. The SWCNT, defect, and low conductivity regions were set to conductivities of 3333, 309, and 2500 S/m, respectively. Forward problems were solved to obtain potential data using the simulation models. Then, the conductivity distribution was reconstructed using an ML model trained on a dataset with a defect conductivity of 309 S/m.

Figure 5b shows a schematic diagram of the estimation method. The ideal defect position and the reconstructed defect position in polar coordinates are denoted as $\:\left({r}_{0},{\theta\:}_{0}\right)$ and $\:\left(r,\theta\:\right)$, respectivily. The deviations $\:\varDelta\:r=r-{r}_{0}\:$and $\:\varDelta\:\theta\:=\theta\:-{\theta\:}_{0}$ were estimated.

Figure 5c and d show $\:\varDelta\:r\:$and $\:\varDelta\:\theta\:$ as a function of electrode number with contact resistance. The dotted line $\:\left(\varDelta\:r=-0.13\:\text{mm},\:\varDelta\:\theta\:=4.46^\circ\:\right)$ represents the estimated reconstruction results using the ideal model, which did not have contact resistance. It has been reported that ML based reconstruction cannot eliminate all deviations, even for input data included in the training dataset^12^.

Compared to the ideal case without contact resistance $\:\left(\varDelta\:r=-0.13\:\text{mm},\:\varDelta\:\theta\:=4.46^\circ\:\right)$ in Fig. 5c, $\:\varDelta\:r$ increased in all cases. This result indicates that the presence of contact resistance caused the reconstructed defect to shift outward. Notably, when contact resistance was present at electrode numbers (6,7,8), the displacement increased, with $\:\varDelta\:r$ increasing by 0.39 mm compared to the ideal case. Based on Fig. 5d, it was shown that contact resistance induces a rotation in the position of the reconstructed defect. In particular, compared to the reconstructed image in the ideal case without contact resistance, the defect position rotated significantly, shifting upward by 9.12° when contact resistance was present at electrode numbers (8,1,2) and downward by 20.75° when present at electrode numbers (4,5,6). When contact resistance was symmetrically distributed around electrode 3 (at electrodes 2, 3, and 4) and electrode 7 (at electrodes 6, 7, and 8), the defect position rotated only slightly, upward by 1.49° and downward by 0.44°, respectively. These results indicate that contact resistance causes the reconstructed defect to shift outward and rotate toward the electrodes with lower conductivity, which is detected as a defect.

**Fig. 6:**
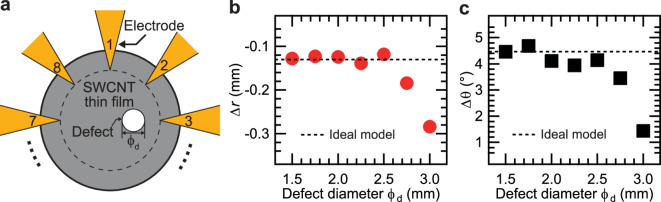
(**a**) Schematic illustration of the simulation model with resized defects. (**b**) $\:\varDelta\:r$ and (**c**) $\:\varDelta\:\theta\:$ as functions of the defect diameter $\:{\phi\:}_{\text{d}}$.

The third factor is the enlargement of defects caused by plasma penetration between the metal mask and the SWCNT thin film during plasma irradiation. Figure 6a shows one of the simulation models. The conductivity of SWCNT and defect were 3333 S/m and 309 S/m, respectively. The defect was placed 3.5 mm to the right of the substrate center. The SWCNT had a diameter of 14 mm, and the defect diameter $\:{\phi\:}_{\text{d}}$ was varied from 1.5 to 3.0 mm. The forward problem was solved using this simulation model to obtain potential data. The conductivity distribution was reconstructed using a ML model trained on a dataset with a defect conductivity of 309 S/m.

Figure 6b and c show $\:\varDelta\:r$ and $\:\varDelta\:\theta\:$ as functions of the defect diameter $\:{\phi\:}_{\text{d}}$. When $\:{\phi\:}_{\text{d}}$ was 3.0, compared to the estimated result of ideal value $\:{\phi\:}_{\text{d}}=1.5\:\text{mm}$, where the deviations were $\:\varDelta\:r=-0.13\:\text{m}\text{m}$ and $\:\varDelta\:\theta\:=4.46^\circ\:$, both $\:\varDelta\:r$ and $\:\varDelta\:\theta\:$ decreased significantly, by 122% and 67%, respectively. This can be attributed to the limited number of training datasets near the substrate edge, resulting in insufficient training dataset for the ML model. The experimentally reconstructed images showed that defects shifted inward by approximately 1 mm, suggesting that defects generated by plasma irradiation had expanded beyond their ideal size.

We analyzed which of the three factors described above contributed to the positional changes of the defects in the reconstructed images shown in Fig. 3a and d.

**Fig. 7:**
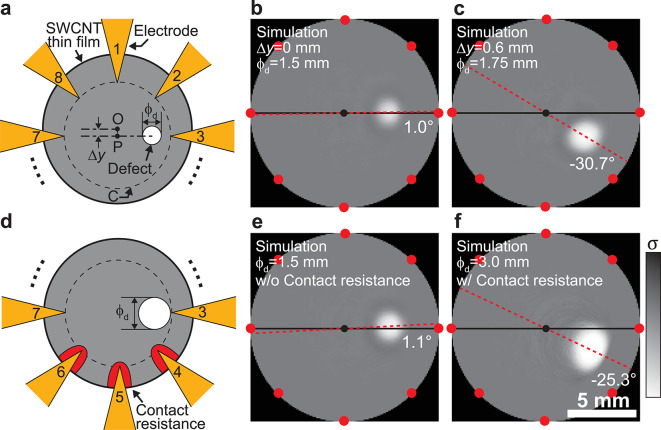
A schematic illustration of a model simulating a sample with a defect fabricated using (**a**) 10 W and (**d**) 40 W plasma irradiation power. Reconstructed images of the ideal model for plasma irradiation powers of (**b**) 10 W and (**e**) 40 W. Reconstructed images using potential data obtained from the model in (**c**) Fig. 7a and (**f**) Fig. 7d.

Figure 7a shows a schematic illustration of one of the simulation models. The center of the SWCNT thin film was defined as point O, and the center of the circle C, which connects the vertices of the triangular electrodes, was defined as point P. The distance between point O and point P was defined as $\:\varDelta\:y$. A defect was fabricated along the line connecting electrode 3 and electrode 7. The diameter of the SWCNT thin film was set to 14 mm, and the diameter of the defect was denoted as $\:{\phi\:}_{\text{d}}$. The conductivities of the SWCNT thin film and the defect fabricated with a 10 W plasma irradiation power were set to 3333 and 1125 S/m, respectively. The conductivity distribution was reconstructed using a ML model trained on a dataset with a defect conductivity of 1125 S/m.

Figure 7b and c show the reconstructed conductivity distributions for conditions of $\:\varDelta\:y=0\text{\:mm}$ and $\:{\phi\:}_{\text{d}}=1.5\:\text{mm}$, and $\:\varDelta\:y=0.6\text{\:mm}$ and $\:{\phi\:}_{\text{d}}=1.75\:\text{mm}$, respectively. In Fig. 7c, $\:\varDelta\:y$ was set to 0.6 mm to simulate the actual sample. The deviations in the reconstructed defect in Fig. 7b and c were $\:\varDelta\:r=-0.3\:\text{m}\text{m}$ and $\:\varDelta\:\theta\:=1.0^\circ\:$, and $\:\varDelta\:r=-0.1\:\text{m}\text{m}$ and $\:\varDelta\:\theta\:=-30.7^\circ\:$, respectively. These results suggest that the main factor causing the defect displacement observed in the reconstructed image of Fig. 3a is the misalignment between the center of the circular SWCNT film and the center of the line connecting electrodes 3 and 7.

Figure 7d shows a schematic illustration of one of the simulation models. The diameter of the SWCNT thin film was set to 14 mm, and the diameter of the defect was denoted as $\:{\phi\:}_{\text{d}}$. A low-conductivity region, simulating the red colored contact resistance, was set near electrodes 4, 5, and 6. The conductivities of the SWCNT thin film, the defect fabricated with a 40 W plasma irradiation power, and the low-conductivity region simulating the contact resistance were set to 3333, 309, and 1000 S/m, respectively. The conductivity distribution was reconstructed using a ML model trained on a dataset with a defect conductivity of 309 S/m.

The reconstructed results using potential data obtained from a simulation model without contact resistance and $\:{\phi\:}_{\text{d}}=1.5\:\text{mm}$ are shown in Fig. 7e, while the results using a model with contact resistance and $\:{\phi\:}_{\text{d}}=3.0\:\text{mm}$ are shown in Fig. 7f. The deviations in the reconstructed defect of Fig. 7e and f were $\:\varDelta\:r=-0.2\:\text{m}\text{m}$ and $\:\varDelta\:\theta\:=1.1^\circ\:$, and $\:\varDelta\:r=-0.2\:\text{m}\text{m}$ and $\:\varDelta\:\theta\:=-25.3^\circ\:$, respectively. Based on the actual sample, corresponding to Fig. 3d, no misalignment was observed between the center of the circular SWCNT film and the center of the line connecting electrodes 3 and 7. Therefore, the main factors contributing to the defect displacement are considered to be contact resistance and the enlargement of defects.

## Conclusion

In this study, we extend the application of ERT to visualize disorder distribution in transparent SWCNT thin films. Based on the experimentally established correlation between disorder and resistance, we successfully converted ERT-reconstructed conductivity distributions into disorder maps. As a result, even a defect region with an *I*_G_/*I*_D_ ratio of approximately 15, which is only slightly lower than the pristine region (*I*_G_/*I*_D_ ~ 18), was clearly visualized corresponding to a minimum detectable *R*/*R*_0_ of 1.59. Furthermore, in a sample containing two defect regions introduced by plasma irradiation at 20 and 40 W, the ERT images effectively distinguished both regions, despite their *I*_G_/*I*_D_ ratios differing by only 1.1. This demonstrates that the proposed method enables the visualization of subtle variations in disorder across the SWCNT thin film.

However, for single-defect experimental samples, the average angular deviation was − 22.8°, and the average radial offset was − 1.6 mm, indicating that the defect was visualized with a slight inward shift and a clockwise rotation relative to the ideal position. We conducted simulations to investigate positional deviations in the reconstructed ERT images and identified three contributing factors: misalignment between the film and electrodes, contact resistance variations, and expansion of the irradiated area due to plasma penetration beneath the mask. These findings underscore the capability of ERT as a non-destructive and sensitive technique for assessing the structural quality of conductive thin films.

In future studies, the proposed ERT technique could be extended to in-situ monitoring of SWCNT-based devices during fabrication or operation, enabling real-time defect detection and quality assurance. Furthermore, this methodology could be adapted for other types of conductive thin films, such as graphene or metal nanowire networks, which also require large-area non-destructive assessment. Integration of ERT with roll-to-roll manufacturing systems may further enhance its potential for industrial application in flexible electronics and optoelectronic devices.

Although the current configuration using vacuum-deposited gold electrodes is not yet suitable for large-scale manufacturing, the ERT concept can be implemented by integrating electrodes into embedded layers within the device structure or using probe-based inline inspection. Future work will focus on developing simplified electrode formation processes and inline-compatible measurement systems to realize practical manufacturing applications.

## Supplementary Information

Below is the link to the electronic supplementary material.

Supplementary Material 1

## Data Availability

The datasets used and/or analyzed during the current study are available from the corresponding author on reasonable request.
