# Supplementary material for: Mapping defect distribution in transparent single-walled carbon nanotube film with electrical resistance tomography
Source: Sci Rep. 2025 Dec 18;16:470. doi: 10.1038/s41598-025-29839-w (PMC12775047; doi:10.1038/s41598-025-29839-w)
Supplement: Supplementary file 1 — Supplementary Material 1 [file 41598_2025_29839_MOESM1_ESM.docx]

**Supplementary information**

**Mapping defect distribution in transparent single-walled carbon nanotube film with electrical resistance tomography**

Keiya Minakawa, Taiki Nakada, Reiji Kaneko, and Takashi Ikuno^*^

Department of Applied Electronics, Graduate School of Advanced Engineering, Tokyo University of Science, Katsushika, Tokyo 125-8585, Japan

*tikuno@rs.tus.ac.jp

Figure S1 shows the reconstruction results for circular, square, and triangular defects. The ML-based ERT model trained with datasets containing multiple geometries successfully visualized all three defect shapes, demonstrating that the framework can be extended to non-circular geometries when appropriate training data are provided.

**Figure S1.** Reconstructed conductivity images of (a) model and (b) ML-based ERT reconstructions for circular, square, and triangular defects.


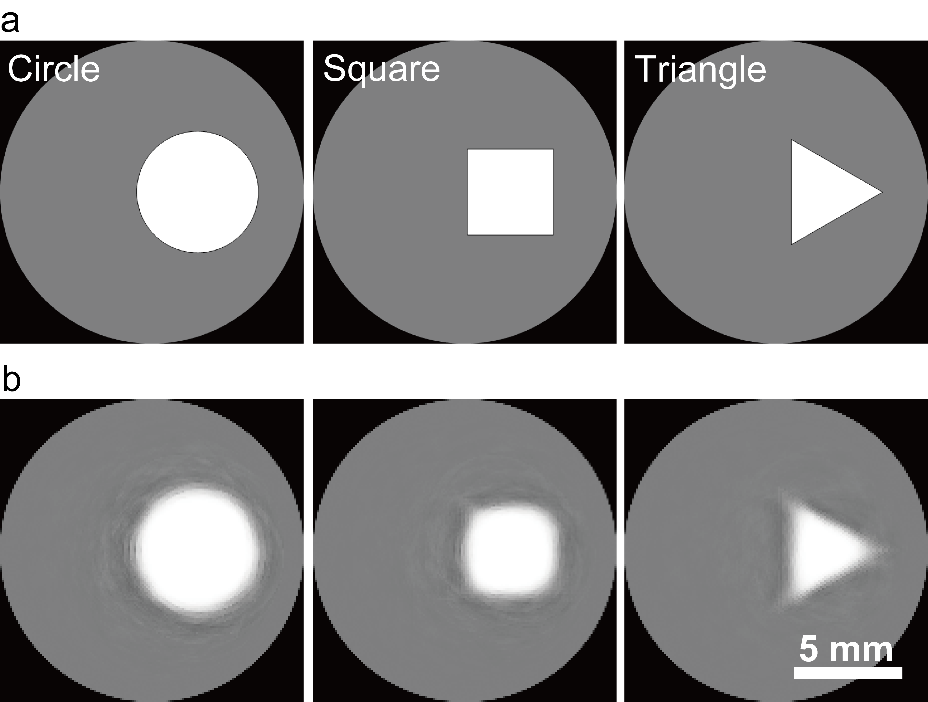


Figure S2 shows the optical reflectance of the samples as a function of light wavelength.

**Figure S2.** Optical reflectance spectra of the fabricated SWNT thin film.


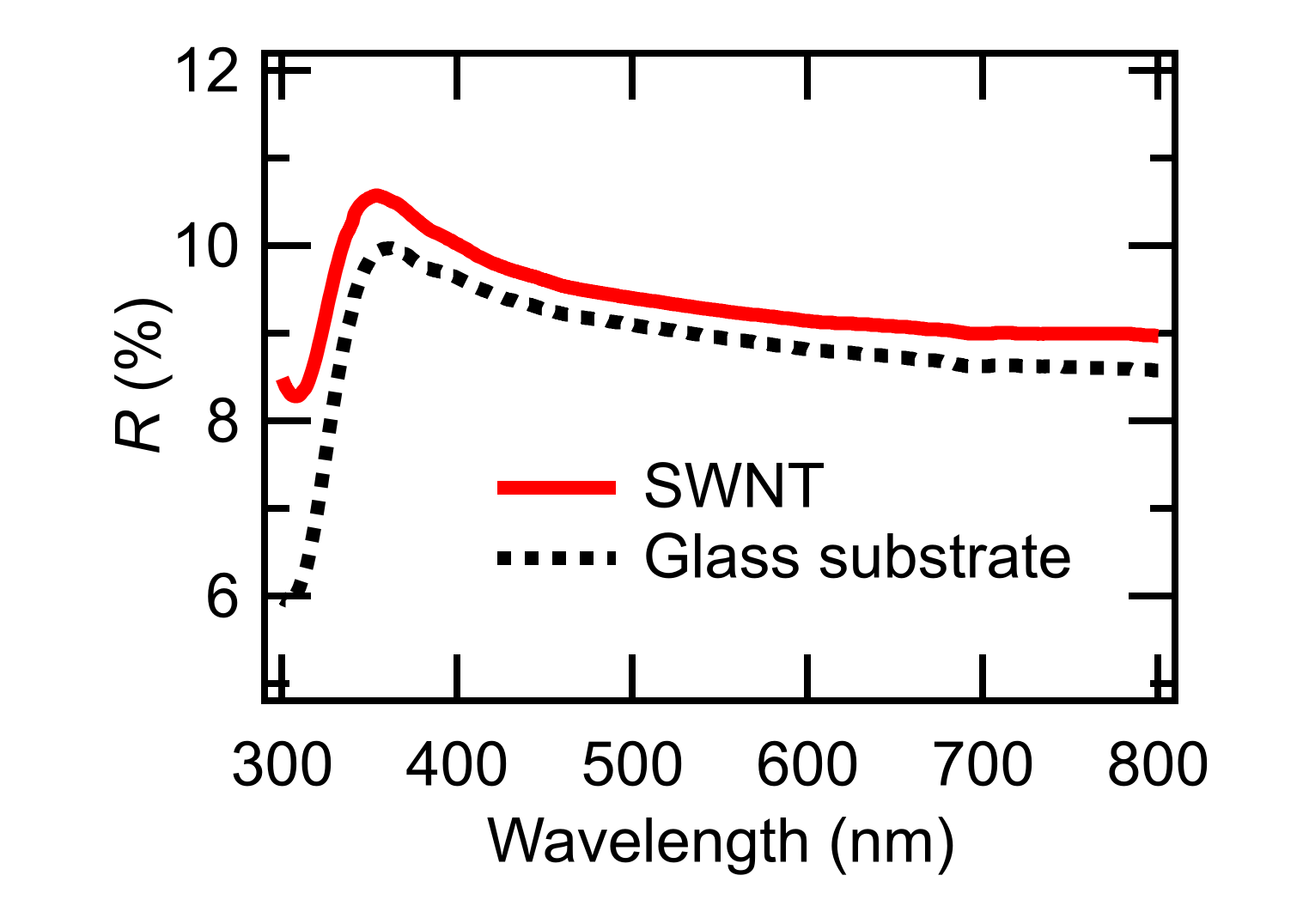


As shown in the simulated spectra (Fig. S3a), the difference in the highlighted region gradually decreased as the defect diameter increased from 0.5 mm to 3.5 mm, reaching −0.10 for the 3.5 mm defect. In the experimental spectra (Fig. S3b), the differences for plasma irradiation powers of 40, 30, 20, and 10 W were −0.22, −0.28, −0.15, and −0.19, respectively. These differences in the experimental data suggest that the reconstructed defect regions appeared larger than the actual mask openings.

**Figure S3.** Comparison between measured and simulated normalized voltage difference spectra. (a) Simulated spectra for defects with different diameters (0.5, 1.5, 2.5, and 3.5 mm). (b) Measured spectra for SWCNT thin films subjected to plasma irradiation at 10, 20, 30, and 40 W. The highlighted regions indicate characteristic variations in potential difference corresponding to defect size changes.


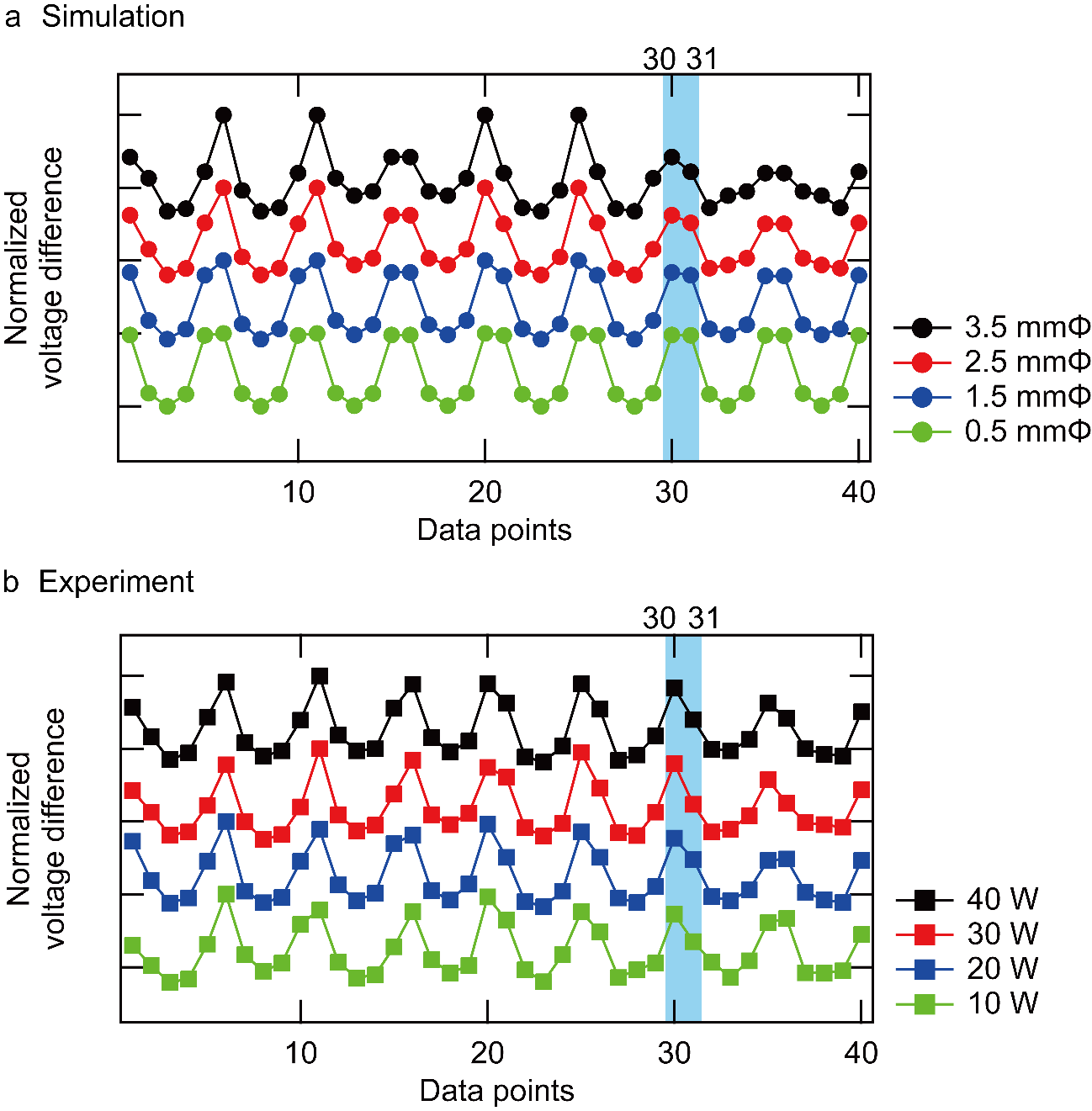


The first factor of positional deviations is the misalignment between the center of SWNT thin film and the center of the line connecting electrodes 3 and 7. To analyze the effect of this misalignment, forward problems were solved using the simulation model shown in Fig. S4a, and the corresponding conductivity distributions were reconstructed. Dotted lines connect the vertices of the electrodes. The black line, with its center at $\left( x,y \right)$, represents the ideal electrode placement. In contrast, in the experimental line shown in red, the electrodes are shifted, with the center at $\left( x_{0},y_{0} \right).$ Defects were fabricated on the line connecting electrodes 3 and 7. The offsets, defined as $\Delta x=x-x_{0}$ and $\Delta y=y-y_{0}$​, were varied from −1 to 1 mm in 0.5 mm steps, and the corresponding potential spectra were obtained. The conductivities of the defect and substrate were set to 309 and 3333 S/m. The resistance distribution image was reconstructed using an ML model trained on a dataset with defect conductivity of 309 S/m.

Figure S4. (a) Schematic illustration of the simulation model and (b) the defect position estimation method. (c) $\Delta r$ and (d) $\Delta\theta$ as functions of $\Delta x$ and $\Delta y$.


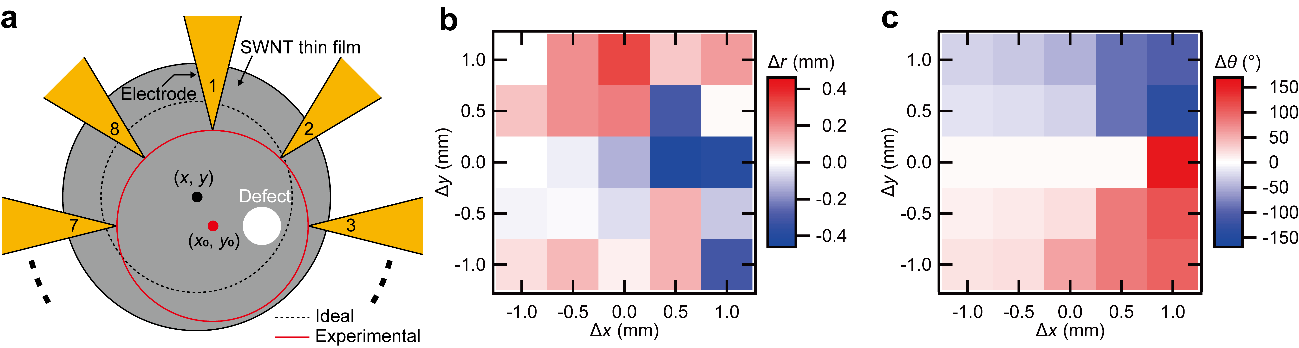


Using the metrics shown in fig.5b, the positional error of the reconstructed defect was estimated. The defect position in the reconstructed image was expressed in polar coordinates. The ideal defect position and the reconstructed defect position are denoted as $\left( r_{0},\theta_{0} \right)$ and $\left( r,\theta\right)$. The errors $\Delta r=r-r_{0}$and $\Delta\theta=\theta-\theta_{0}$ were estimated.

The relationships of $\Delta r$ and $\Delta\theta$ as functions of $\Delta x$ and $\Delta y$ are shown in Figs. S2b and S2c. There was no clear pattern in $\Delta r$. While, When $\Delta y>0$, the defect shifted relatively downward, resulting in $\Delta\theta<0$. Conversely, when $\Delta y<0$, the defect shifted relatively upward, resulting in $\Delta\theta>0$. These results indicate that the reconstructed defect position changes with $\Delta y$.
